# Knowledge, Attitudes, and Practices towards Antibiotics, Antimicrobial Resistance, and Antibiotic Consumption in the Population of Kazakhstan

**DOI:** 10.3390/antibiotics13080718

**Published:** 2024-07-31

**Authors:** Yuliya Semenova, Laura Kassym, Assiya Kussainova, Ainur Aimurziyeva, Larissa Makalkina, Andrey Avdeyev, Aizhan Yessmagambetova, Manar Smagul, Bibigul Aubakirova, Zaure Akhmetova, Ademi Yergaliyeva, Lisa Lim

**Affiliations:** 1 School of Medicine, Nazarbayev University, Astana 010000, Kazakhstan; laura.kassym@nu.edu.kz (L.K.); a.kussainova@nu.edu.kz (A.K.); 2 School of Sciences and Humanities, Nazarbayev University, Astana 010000, Kazakhstan; ainur.aimurziyeva@nu.edu.kz; 3 Department of Clinical Pharmacology, Astana Medical University, Astana 010000, Kazakhstan; makalkina.l@amu.kz; 4 Medical Center Hospital of the President’s Affairs Administration of the Republic of Kazakhstan, Astana 010000, Kazakhstan; avdeyev@bmc.mcudp.kz; 5 Ministry of Health of the Republic of Kazakhstan, Astana 010000, Kazakhstan; yesmagambetovaa@dsm.gov.kz (A.Y.); z.akhmetova@dsm.gov.kz (Z.A.); 6 National Center of Public Healthcare, Astana 010000, Kazakhstan; manar.smagul@gmail.com (M.S.); ergalievaadema00@gmail.com (A.Y.); 7 WHO Country Office in Kazakhstan, Astana 010000, Kazakhstan; aubakirovab@who.int; 8 Graduate School of Public Policy, Nazarbayev University Astana 010000, Kazakhstan; lisa.lim@nu.edu.kz

**Keywords:** knowledge, attitudes and practice, antimicrobial resistance, antibiotics, Kazakhstan

## Abstract

During the COVID-19 pandemic, a ban on inspections of small businesses, including pharmacies, was imposed in Kazakhstan, which relaxed law enforcement efforts regarding the prohibition of over-the-counter antibiotic (AB) sales. This study aimed to investigate how this affected the knowledge, attitudes, and practices (KAP) related to AB and antimicrobial resistance (AMR), as well as to assess actual AB consumption at the community level. The study comprised two cross-sectional sub-studies: the first involved a KAP survey conducted in 2022 and 2024, utilizing the Special Eurobarometer questionnaire on AMR. The second sub-study analyzed AB consumption in 2021 and 2023, measured in defined daily doses per 1000 inhabitants. Results revealed an increase in the percentage of individuals reporting receipt of information about ABs and AMR in the past year (37.3% in 2022 vs. 52.9% in 2024, *p* < 0.001) and an increase in the percentage of individuals reporting AB use in the past year (49.0% in 2022 vs. 54.0% in 2024, *p* = 0.056). The most consumed ABs were from the Watch group, with azithromycin and ceftriaxone ranking highest. These findings support the hypothesis that the relaxation of law enforcement contributed to an increase in AB consumption and emphasize the need for public health policies to address this issue.

## 1. Introduction

The World Health Organization (WHO) considers antimicrobial resistance (AMR) to be among the top 10 threats to global health. As of 2019, AMR caused 1.27 million deaths per year, and this number is predicted to rise to 10 million deaths annually by 2050, comparable to the number of deaths from cancer [1]. Antibiotics constitute the largest class of antimicrobial drugs, and antibiotic resistance is becoming an alarming trend, leading to changes in treatment strategies for many bacterial diseases and reducing treatment efficacy overall [2]. In response to this critical issue, antimicrobial stewardship (AMS) was developed as a coordinated approach to optimize the use of antibiotics; promote the appropriate selection, dosage, and duration of antimicrobial treatment; and minimize the emergence of resistance [3].

AMS programs are designed to enhance patient outcomes while reducing adverse effects and limiting the spread of resistant bacteria. A key component of AMS is the education and training of healthcare providers on the principles of responsible antibiotic use [4]. This includes encouraging them to prescribe antibiotics only when necessary and to select the most appropriate agents based on local resistance patterns and individual patient needs [5]. Another important element is public health initiatives aimed at raising awareness about the dangers of antibiotic misuse among the general population [2]. These initiatives include campaigns to educate people on the importance of obtaining prescriptions for antibiotics, avoiding self-medication, and refraining from using leftover antibiotics, among other measures [6].

The Republic of Kazakhstan (hereafter referred to as Kazakhstan) is a central Asian country that gained its independence in 1991. The healthcare system model originates from the Semashko model of the Soviet period [7]. The country has implemented several aspects of AMS by establishing a hospital AMS program in selected hospitals, participating in “World Antibiotic Awareness Week”, and introducing legislation prohibiting over-the-counter (OTC) sales of antibiotics [8]. This legislation was adopted in 2002 as an order of the Ministry of Health (MoH) but was not implemented for many years until 2016, when it was reinforced [9]. In the subsequent period, there was a series of inspections of pharmacies, and pharmacies selling antibiotics OTC were fined [10]. The emergence of the COVID-19 pandemic resulted in a profound shortage of healthcare resources and was accompanied by a ban on inspections of small businesses, including pharmacies [11]. This led to a relaxation of the law enforcement efforts related to the prohibition of OTC sales of antibiotics.

Currently, there is a lack of studies investigating the implementation of AMS programs in Kazakhstan. Similarly, there is a lack of studies investigating the impact of the COVID-19 pandemic on the consumption of antibiotics. This study aims to fill this gap by investigating two specific subtopics: knowledge, attitudes, and practices (KAP) towards antibiotics and AMR among the general population and the actual consumption of antibiotics at the community level during and after the pandemic. This research is crucial to determine whether the relaxation of law enforcement on the prohibition of OTC antibiotic sales during the COVID-19 pandemic led to irresponsible antibiotic use. Furthermore, the findings will inform the development and implementation of AMS strategies in Kazakhstan.

## 2. Results

### 2.1. Survey on Knowledge, Attitudes, and Practices Regarding Antibiotics and Antimicrobial Resistance

The sociodemographic details of the study participants are presented in Table 1. Overall, the study sample accurately represents the country’s population [12]. There were no significant differences between the respondents interviewed in 2022 and 2024 except for financial wellbeing and age at completion of education.

The knowledge related to antibiotics and AMR is presented in Table 2. Overall, 60.3% of respondents believed that antibiotics kill viruses. This was the only significant difference between the groups interviewed in 2022 and 2024, with fewer people in 2024 holding this belief (55.8% vs. 64.7%). A higher proportion, 67.5%, believed that antibiotics are effective against colds. However, 63.6% of respondents were aware of AMR, and 58.7% knew that taking antibiotics is associated with side effects. Additionally, 78.3% of respondents believed that antibiotics should only be discontinued once all of the prescribed antibiotics are taken as directed by the doctor.

Overall, 45.1% of respondents recalled receiving information about avoiding unnecessary antibiotic use in the past 12 months, with this proportion being higher in 2024 compared to 2022 (52.9% vs. 37.3%, respectively). The primary source of this information was the Internet or online social networks, and its significance increased in 2024 compared to 2022 (53.1% vs. 40.3%, respectively). More than half (59.6%) of the respondents admitted that this information changed their views on using antibiotics. Most respondents expressed a desire for more information about antibiotics and AMR in the future. The most preferred topics for additional information were how to use antibiotics (39.0%), AMR (36.3%), and medical conditions for which antibiotics are used (32.5%). A doctor was considered the most trustworthy source of information (66.4%), followed by Internet resources (53.1%): an official health-related website, a health-related personal blog, another health-related website, or online social networks (Table 3).

The proportion of respondents who reported taking oral antibiotics in the past 12 months increased in 2024 compared to 2022 (54.0% vs. 49.0%, respectively), although this change was not statistically significant. Respiratory tract infections were the most common reason for taking antibiotics, despite many of these infections typically being caused by viruses. More than a quarter of respondents (26.3%) used antibiotics to treat fever, and 18.6% used them to treat headaches, with an increase in these proportions observed in 2024. In addition, more respondents reported that the COVID-19 pandemic decreased their need for antibiotics (due to strengthened personal protective measures and/or falling ill less often during the containment period) than those who believed it increased the need for antibiotics or had no impact (53.0% vs. 39.9%, respectively) (Table 4).

### 2.2. Study on the Consumption of Antibiotics at the Community Level

The consumption of antibiotics, expressed in DDDs per 1000 inhabitants, increased in 2023 compared to 2021 (22.5491 vs. 22.21783, respectively). However, it declined in terms of packages per inhabitant per year (5.073366 vs. 5.771523, respectively). Azithromycin was the most consumed antibiotic in both 2021 and 2023, with 3.455283 and 3.336897 DDDs per 1000 inhabitants, respectively, followed by ceftriaxone (2.571002 and 2.037079 DDDs per 1000 inhabitants, respectively) and amoxicillin with clavulanic acid (1.553602 and 2.236595 DDDs per 1000 inhabitants, respectively). Ceftriaxone also led in consumption expressed in packages per inhabitant per year, with 1.873313 packages in 2021 and 1.45889 packages in 2023 (Table 5).

**Figure 1:**
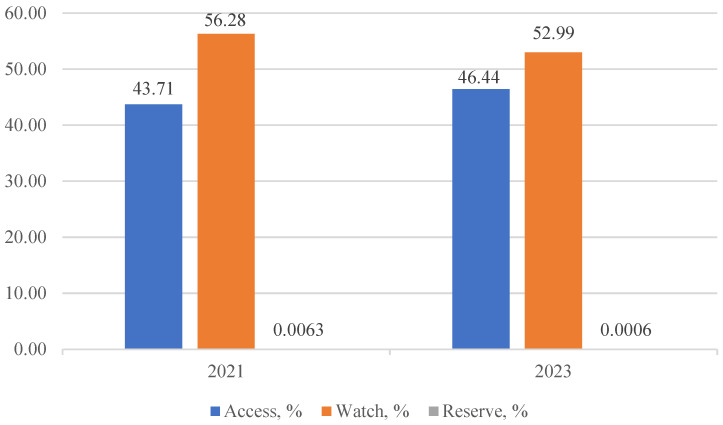
Consumption of antibiotics by AWaRe categories in 2021 and 2023, %.

The consumption of antibiotics from the Watch group predominated in both 2021 and 2023 (56.28% vs. 52.99%, respectively), while the consumption of antibiotics from the Access group was 43.71% in 2021 and 46.44% in 2023. This contradicts the WHO recommendation that at least 60% of antibiotics consumed should belong to the Access group. Fortunately, the consumption of antibiotics from the Reserve group was well below the recommended “less than 1%” [13], constituting 0.0063% in 2021 and 0.0006% in 2023 (Figure 1).

## 3. Discussion

This study aimed to investigate the KAP regarding antibiotics and AMR among the Kazakhstani population, as well as the actual consumption of antibiotics in the community sector during two periods: during the COVID-19 pandemic and after it. The results indicated a significant post-pandemic improvement in awareness that antibiotics do not kill viruses. In addition, there was a significant increase in the percentage of people who reported receiving information about antibiotics and AMR within the past 12 months, rising from 37.3% during the pandemic to 52.9% after the pandemic. The sources of this information also changed, with the Internet and social networks becoming more important after the pandemic. Respondents expressed a preference for continuing to receive information through these channels in the future. The share of people who reported antibiotic intake within the past 12 months increased insignificantly, a trend supported by the data on DDDs per 1000 inhabitants, which also increased during the same period. Antibiotics belonging to the Watch group were the most consumed, with azithromycin and ceftriaxone holding the first and second positions, respectively. Watch group antibiotics led community consumption in both 2021 and 2023, although their share declined over time. The findings of this study warrant a detailed discussion to understand their implications and to inform future AMS strategies.

The extent to which the existing AMS strategies contributed to improved knowledge about antibiotics in this study is debatable. The fact that more people knew that antibiotics do not kill viruses could be attributed to the COVID-19 pandemic itself, caused by a viral pathogen, and the circulating information in mass media about viruses and methods of their treatment [14]. Amato et al. made a similar observation regarding significantly improved knowledge among Ecuadorian parents about the fact that antibiotics do not kill viruses, which increased from 27% pre-pandemic to 38% during the pandemic. The authors also did not attribute this change to the existing AMS program [15]. Overall, 63.6% of respondents were aware that unnecessary use of antibiotics makes them ineffective. In addition, more than half of the respondents were aware of the side effects associated with antibiotic intake, particularly diarrhea, which can be attributed to the widespread advertisement of probiotics frequently circulating in Kazakhstani mass media [16].

This study provided valuable insights into the sources of information on antibiotics and AMR currently used by the general population in Kazakhstan and highlighted the preferred sources for obtaining trustworthy information, which is crucial for the effective delivery of AMS programs. The Internet and online social networks were identified as major sources of information, exceeding even doctors’ advice (47.8% vs. 43.4%, respectively). Internet and social networks are also perceived as trustworthy sources of information on antibiotics and AMR, with 53.1% of respondents preferring this path. This observation could be attributed to the fact that Kazakhstani society demonstrates high levels of technocratic optimism, with the Internet being the preferred communication channel [17]. However, an even larger proportion of respondents (66.4%) considered a doctor to be the most trustworthy source of information, emphasizing the importance of involving doctors in AMS strategies.

This study also identified the preferred topics for receiving additional information, with AMR, proper antibiotic usage, and medical conditions for which antibiotics are used being the most frequently mentioned (36.6%, 39.0%, and 32.5%, respectively). Notably, there was an increase in the proportion of people indicating that they used antibiotics to treat fever (from 22.5% during the pandemic to 29.7% post-pandemic) and headache (from 14.0% to 22.8%). This highlights the need for public campaigns aimed at improving awareness about the appropriate indications for antibiotic use.

Based on this study, Kazakhstan falls into the Watch group in terms of antibiotic consumption. These findings are consistent with earlier research reporting the structure of antibiotic consumption in the hospital sector. For instance, an earlier study by Zhussupova et al. found that Watch group antibiotics accounted for 68% of antibiotic consumption in 2019, with an upward trend [8]. It should be noted that the hospital population has an increased need for antibiotics compared to the community population, thus justifying a higher share of Watch group antibiotics [18]. However, the fact that Watch group antibiotics hold a large share at the community level requires consideration and calls for the need to implement an AMS strategy to bring the share of Watch group antibiotics within the recommended threshold of below 40% and ensure that the consumption of Access group antibiotics is at least 60% [19].

This study has several limitations, with the major one being that it is a single-city survey. However, it was conducted in the largest city of Kazakhstan, which accounts for more than 10% of the country’s population and attracts a significant number of visitors from different parts of Kazakhstan, potentially contributing to the overall sample. Another limitation is that data were collected during different seasons, which may impose seasonal effects. Nevertheless, the periods of October–November and March–April share many similarities in terms of average temperatures, precipitation levels, and the number of clear and windy days, making them comparable [20]. In addition, all respondents were interviewed on the street upon their exit from public places. This setting may affect the accuracy of their responses due to numerous distractions and a tendency for people to hurry, especially in unfavorable weather conditions. However, the survey questionnaire was short and straightforward, minimizing the need for detailed responses if a previous answer was negative.

This study also has obvious strengths. By repeating the data collection twice, it provides insights into changes in KAP over time. Another significant strength is that it is the first study to report on antibiotic consumption at the community level in Kazakhstan using a reliable source of information, providing comparisons during and after the pandemic. Future research should expand the geographic area of studies to include other parts of Kazakhstan. In addition, there is a need for the implementation of AMS programs at the community level with subsequent evaluations and adjustments to enhance performance.

## 4. Materials and Methods

This study consists of two separate cross-sectional sub-studies. The first sub-study is a survey examining the KAP towards antibiotics and AMR among the population of Kazakhstan, conducted in two phases: in 2022 (during the COVID-19 pandemic) and in 2024 (post-pandemic). The second sub-study comprises the analysis of data on antibiotic consumption at the community level in 2021 and 2023, given that the questions in the KAP questionnaire referred to the past 12 months, i.e., 2021 and 2023. Taken together, these sub-studies enable the characterization of the KAP of the general population of Kazakhstan regarding antibiotics and AMR, as well as the actual consumption of antibiotics in the community health sector.

### 4.1. Study Site

Almaty, located in the southeast of Kazakhstan, was selected as the site for data collection. As the former capital and home to over 10% of the country’s population, Almaty is Kazakhstan’s major commercial, financial, educational, and cultural center. The city’s large and diverse labor market attracts people from across the country, and its extensive network of educational facilities draws students nationwide [21]. Given these unique characteristics, it was postulated that this study site would enable a broad representation of the KAP related to antibiotics and AMR.

### 4.2. Survey on Knowledge, Attitudes, and Practices Regarding Antibiotics and Antimicrobial Resistance

#### 4.2.1. Study Design and Participants

This cross-sectional study collected data at two distinct time periods: between 12 October and 17 November 2022 (during the COVID-19 pandemic), and between 13 March and 18 April 2024 (post-pandemic). Due to Almaty’s temperately continental climate, these periods are characterized by moderately cool weather with infrequent rain [20]. The aim was to reveal differences in the patterns of KAP regarding antibiotics, as well as their consumption. Face-to-face interviews were conducted with individuals aged 18 years and older upon their exit from public places such as subway/bus/railroad stations, shopping malls, universities, and healthcare facilities/pharmacies. The contribution of each data collection site to the overall sample size did not exceed 10%.

The sample size calculation was performed using the StatCalc function of Epi Info software, version 7.2.5.0. A population survey was selected with the following inputs: the population size of Almaty city (approximately 2,000,000 people) [12], an expected frequency of knowledge of antibiotics and AMR of 18% [16], a 5% acceptable margin of error, and a design effect of 2.0. For a 95% confidence interval (CI), the calculated sample size was 454 people. Systematic random sampling was utilized for data collection, with every second adult individual exiting the selected site approached and invited to participate in the study. Potential respondents were provided with information on the study’s aims and procedures. In 2022, 650 individuals were approached, of whom 553 provided informed consent, resulting in an 85.1% response rate. In 2024, 650 individuals were also approached, with 550 granting informed consent, yielding an 84.6% response rate. Data collection was conducted anonymously following the receipt of informed consent.

#### 4.2.2. The Questionnaire

This study utilized the Special Eurobarometer questionnaire Number 522, “Antimicrobial resistance” [22]. This questionnaire is structured into two subsections. The first subsection collects information on the socioeconomic characteristics of the study participants, including gender, age group, age at completion of education, socio-professional category, and financial status, evaluated in terms of difficulty in paying bills. The second subsection gathers information on the KAP related to antibiotic consumption over the past 12 months and AMR. Comprising 16 questions, this section covers a broad spectrum of topics: specific knowledge about antibiotics and AMR (e.g., the ability of antibiotics to kill viruses, their effectiveness against the common cold, side effects, the duration of antibacterial therapy, and their ability to cause resistance), information received and preferred regarding antibiotic consumption and AMR (sources of information, their impact on respondents’ views and plans, and preferred and trustworthy information sources), and practices (e.g., the last course of antibiotics taken, indications for use, and methods of obtaining antibiotics). Two specific questions evaluate the impact of the COVID-19 pandemic on access to and the consumption of antibiotics. The same set of questions was used to collect data for both phase one (2022) and phase two (2024) of the present study.

#### 4.2.3. Statistical Analysis

The survey data were analyzed using the Statistical Package for Social Sciences (SPSS) software, version 24. Since all generated variables were categorical, they were presented as a number (n) and percentage (%). The variables were disaggregated by the phase of the survey (2022 and 2024) and also presented in total. Pearson’s chi-square test was used to compare the differences between the two survey phases (2022 vs. 2024), with the level of statistical significance set at 0.05.

### 4.3. Study on the Consumption of Antibiotics at the Community Level

#### 4.3.1. Information Source

The database created and maintained by the market research company “Vi-ORTIS” (Almaty, Kazakhstan) served as the source of information on the consumption of antibiotics at the community level. Vi-ORTIS collects information on pharmacy sales in Kazakhstan through various methods, including tracking pharmacies’ procurement of medicines from distributors and their sales to patients. This is facilitated by the free provision of “PharmCenter” software to pharmacies, which is utilized by over 90% of pharmacies in Almaty [23]. All information gathered by Vi-ORTIS is standardized, and a unified report is compiled from all data sources on a monthly basis. During the preparation of this report, data on procurements and sales are compared and consolidated, and data on returns and transfers between different pharmacies are accounted for. More information about the use of Vi-ORTIS data for pharmacoepidemiological research can be found elsewhere [24].

From the Vi-ORTIS database, which is maintained as a web portal, data on systemic antibacterials (J01 code) were downloaded for Almaty city for the periods from 1 January to 31 December 2021 and 1 January to 31 December 2023. These time periods were selected because the survey was conducted in two phases in 2022 and 2024, and the questions referred to the past 12 months. Data on all antibiotics were extracted based on level 5 of the Anatomical Therapeutic Chemical classification (ATC5) and included the product name, active ingredient(s), dosage form, active ingredients per unit dose, route of administration, number of tablets/capsules/sachets/suspensions/ampoules/vials in a package, and the number of packages sold.

#### 4.3.2. Calculating Consumption of Antibiotics

All data extracted from the Vi-ORTIS database were entered into the Excel template of the Global Antimicrobial Resistance and Use Surveillance System for surveillance of AMR and antimicrobial use (GLASS-AMC). This template serves as a foundation for organizing data on antibiotic consumption, calculating consumption based on the Anatomical Therapeutic Chemical/defined daily dose (ATC/DDD) approach, and generating metrics and indicators on antibiotic consumption. The instructions provided in the GLASS Manual on the management of antimicrobial consumption data [25] were strictly followed. Defined daily doses (DDDs) per 1000 inhabitants per day, as well as packages per inhabitant per year, were calculated for each ATC5 code and for the J01 category overall for 2021 and 2023. The population size of Almaty, which was 1,977,011 people in 2021 and 2,211,198 in 2023, was used as the denominator [12].

The WHO’s Access, Watch, Reserve (AWaRe) classification of antibiotics [19] was used to group all ATC5 codes into these three categories. The DDD per 1000 inhabitants per day was calculated for the Access, Watch, and Reserve groups, as well as their proportion in relation to the total DDD per 1000 inhabitants per day for the entire J01 category.

### 4.4. Ethics Statement

The Ethics Committee of the Kazakh National Medical University named after Asfendiyarov granted permission for the study (protocol #1426 dated 29 June 2022).

## 5. Conclusions

The findings of this study support the initial hypothesis that the relaxation of law enforcement on prohibiting OTC antibiotic sales during the COVID-19 pandemic contributed to the increase in antibiotic consumption. This observation is evidenced by the rise in the percentage of individuals reporting antibiotic use in the past 12 months (49.0% in 2022 vs. 54.0% in 2024, *p* = 0.056) and by the growth of antibiotic consumption at the community level, expressed in DDDs per 1000 inhabitants from 22.21783 in 2021 to 22.5491 in 2023. Another noteworthy finding is that Watch group antibiotics were the most consumed at the community level, constituting 56.28% in 2021 and 52.99% in 2023, with azithromycin and ceftriaxone ranking highest. These findings highlight the need for a comprehensive AMS strategy to address this issue.

## Figures and Tables

**Table 1:** Sociodemographic characteristics of study participants.

Characteristics		Total (n = 1103) n (%)	2022 (n = 553) n (%)	2024 (n = 550) n (%)	*p*-Value *
Gender	Male	586 (53.1)	306 (55.3)	280 (50.9)	0.298
	Female	514 (46.6)	246 (44.5)	268 (48.7)	
	Other	3 (0.3)	1 (0.2)	2 (0.4)	
Age group, years	15–24	347 (31.5)	161 (29.1)	186 (33.8)	0.388
	25–39	471 (42.7)	244 (44.1)	227 (41.3)	
	40–54	213 (19.3)	109 (19.7)	104 (18.9)	
	≥55	72 (6.5)	39 (7.1)	33 (6.0)	
Age at completion of education, years	Education is not finished yet	318 (28.8)	137 (24.8)	181 (32.9)	0.013
	≤15	12 (1.1)	4 (0.7)	8 (1.5)	
	16–19	223 (20.2)	119 (21.5)	104 (18.9)	
	≥20	550 (49.9)	293 (53.0)	257 (46.7)	
Socio-professional category	Self-employed	167 (15.1)	88 (15.9)	79 (14.4)	0.115
	Manager	74 (6.7)	39 (7.1)	35 (6.4)	
	Other white collar	300 (27.2)	161 (29.1)	139 (25.3)	
	Blue collar	102 (9.2)	55 (9.9)	47 (8.5)	
	House person	72 (6.5)	40 (7.2)	32 (5.8)	
	Unemployed	51 (4.6)	18 (3.3)	33 (6.0)	
	Retired	40 (3.6)	17 (3.1)	23 (4.2)	
	Student	297 (26.9)	135 (24.4)	162 (29.5)	
Difficulties paying bills	Most of the time	134 (12.1)	51 (9.2)	83 (15.1)	0.007
	From time to time	414 (37.5)	202 (36.5)	212 (38.5)	
	Almost never	305 (27.7)	169 (30.6)	136 (24.7)	
	Never	250 (22.7)	131 (23.7)	119 (21.6)	

* Pearson’s chi-squared test was used to compare the differences between 2022 and 2024.

**Table 2:** Knowledge of respondents about antibiotics and antimicrobial resistance.

Knowledge		Total (n = 1103) n (%)	2022 (n = 553) n (%)	2024 (n = 550) n (%)	*p*-Value *
Antibiotics kill viruses	False	254 (23.0)	105 (19.0)	149 (27.1)	0.003
	True	665 (60.3)	358 (64.7)	307 (55.8)	
	I don’t know	184 (16.7)	90 (16.3)	94 (17.1)	
Antibiotics are effective against colds	False	189 (17.1)	85 (15.4)	104 (18.9)	0.130
	True	745 (67.5)	389 (70.3)	356 (64.7)	
	I don’t know	169 (15.3)	79 (14.3)	90 (16.4)	
Unnecessary use of antibiotics makes them become ineffective	False	160 (14.5)	84 (15.2)	76 (13.8)	0.497
	True	701 (63.6)	342 (61.8)	359 (65.3)	
	I don’t know	242 (21.9)	127 (23.0)	115 (20.9)	
Taking antibiotics often has side effects such as diarrhea	False	182 (16.5)	85 (15.4)	97 (17.6)	0.588
	True	648 (58.7)	328 (59.3)	320 (58.2)	
	I don’t know	273 (24.8)	140 (25.3)	133 (24.2)	
When do you think you should stop taking antibiotics when once you have begun a course of treatment?	When you feel better	166 (15.0)	89 (16.1)	77 (14.0)	0.534
	When you have taken all of the antibiotics as directed by your doctor	864 (78.3)	429 (77.6)	435 (79.1)	
	Don’t know	39 (3.5)	21 (3.8)	18 (3.3)	
	Other	34 (3.1)	14 (2.5)	20 (3.6)	

* Pearson’s chi-squared test was used to compare the differences between 2022 and 2024.

**Table 3:** Attitudes of respondents towards receiving information about antibiotics and antimicrobial resistance.

Attitudes		Total n (%)	2022 n (%)	2024 n (%)	*p*-Value *
Receiving any information about avoiding unnecessary antibiotic use in the past 12 months (n = 1103)	No	491 (44.5)	287 (51.9)	204 (37.1)	<0.001
	Yes	497 (45.1)	206 (37.3)	291 (52.9)	
	I don’t know	115 (10.4)	60 (10.8)	55 (10.0)	
Source of information about avoiding unnecessary antibiotic use (n = 498, multiple answers were permitted)	A doctor	216 (43.4)	112 (54.4)	104 (35.6)	0.001
	A pharmacist	63 (12.7)	28 (13.6)	35 (12.0)	
	Another health professional	84 (16.9)	37 (18.0)	47 (16.1)	
	A family member or friend	182 (36.5)	84 (40.8)	98 (33.6)	
	A TV advertisement	77 (15.5)	47 (22.8)	30 (10.3)	
	Internet or online social networks	238 (47.8)	83 (40.3)	155 (53.1)	
	A leaflet or a poster	33 (6.6)	16 (7.8)	17 (5.8)	
	A newspaper	12 (2.4)	5 (2.4)	7 (2.4)	
	TV news or other programs	50 (10.0)	23 (11.2)	27 (9.2)	
	The radio	7 (1.4)	2 (1.0)	5 (1.7)	
	Other	27 (5.4)	5 (2.4)	22 (7.5)	
	I don’t know	4 (0.8)	3 (1.5)	1 (0.3)	
The information received changed the views on using antibiotics (n = 498)	No	155 (32.4)	41 (22.2)	114 (38.9)	<0.001
	Yes	285 (59.6)	134 (72.4)	151 (51.5)	
	I don’t know	38 (7.9)	10 (5.4)	28 (9.6)	
Plans for the use of antibiotics based on the information received (n = 431, multiple answers were permitted)	A doctor will always be consulted before using antibiotics	202 (46.9)	109 (81.3)	93 (31.3)	<0.001
	Self-medication will be avoided	182 (42.2)	75 (56.0)	107 (36.0)	
	Antibiotics will not be taken without a doctor’s prescription	171 (39.7)	62 (46.3)	109 (36.7)	
	Leftover antibiotics will no longer be kept	43 (10.0)	8 (6.0)	35 (11.8)	
	Leftover antibiotics will no longer be shared with relatives or friends	12 (2.8)	3 (2.2)	9 (3.0)	
	Other	17 (3.9)	1 (0.7)	16 (5.4)	
	None	16 (3.7)	2 (1.5)	14 (4.7)	
	I do not wish to answer	7 (1.6)	1 (0.7)	6 (2.0)	
	I don’t know	1 (0.2)	1 (0.7)	0 (0.0)	
Preferred topics for receiving information, if any (n = 1103, multiple answers were permitted)	Resistance to antibiotics	404 (36.6)	176 (31.8)	228 (41.5)	<0.001
	How to use antibiotics	430 (39.0)	201 (26.3)	229 (41.6)	
	Medical conditions for which antibiotics are used	358 (32.5)	141 (25.5)	217 (39.5)	
	Prescription of antibiotics	293 (26.6)	160 (28.9)	133 (24.2)	
	One Health	192 (17.4)	67 (12.1)	125 (22.7)	
	Other	44 (4.0)	12 (2.2)	32 (5.8)	
	None	78 (7.1)	43 (7.8)	35 (6.4)	
	Refusal to receive information	146 (13.2)	80 (14.5)	66 (12.0)	
	I don’t know	50 (4.5)	18 (3.3)	32 (5.8)	
Information sources to be used to obtain trustworthy information on antibiotics (n = 1103, maximum 3 answers were permitted)	A doctor	732 (66.4)	388 (70.2)	344 (62.5)	<0.001
	A nurse	79 (7.2)	26 (4.7)	53 (9.6)	
	A pharmacy	250 (22.7)	156 (28.2)	94 (17.1)	
	A hospital	299 (27.1)	122 (22.1)	177 (32.2)	
	Another healthcare facility	71 (6.4)	20 (3.6)	51 (9.3)	
	Family or friends	180 (16.3)	85 (15.4)	95 (17.3)	
	An official health-related website	269 (24.4)	94 (17.0)	175 (31.8)	
	A health-related personal blog	65 (5.9)	6 (1.1)	59 (10.7)	
	Another health-related website	72 (6.5)	18 (3.3)	54 (9.8)	
	Online social networks	180 (16.3)	93 (16.8)	87 (15.8)	
	TV	34 (3.1)	14 (2.5)	20 (3.6)	
	Newspapers or magazines	12 (1.1)	3 (0.5)	9 (1.6)	
	The radio	8 (0.7)	2 (0.4)	6 (1.1)	
	Other	20 (1.8)	5 (0.9)	15 (2.7)	
	Refusal to find information	63 (5.7)	31 (5.6)	32 (5.8)	

* Pearson’s chi-squared test was used to compare the differences between 2022 and 2024.

**Table 4:** Practices of antibiotic consumption among survey respondents.

Practices		Total (n = 1103) n (%)	2022 (n = 553) n (%)	2024 (n = 550) n (%)	*p*-Value *
Consumption of oral antibiotics within the past 12 months	No	425 (38.5)	226 (40.9)	199 (36.2)	0.056
	Yes	568 (51.5)	271 (49.0)	297 (54.0)	
	I do not wish to answer	8 (0.7)	7 (1.3)	1 (0.2)	
	I don’t know	102 (9.2)	49 (8.9)	53 (9.6)	
Ways of obtaining the last course of antibiotics used	From a medical prescription	245 (42.8)	114 (42.1)	131 (43.4)	0.125
	Administered by a medical practitioner	71 (12.4)	27 (10.0)	44 (14.6)	
	Use of leftover antibiotics	62 (10.8)	32 (11.8)	30 (9.9)	
	Without prescription from a pharmacy	135 (23.6)	75 (27.7)	60 (19.9)	
	Without prescription from elsewhere	32 (5.6)	12 (4.4)	20 (6.6)	
	I don’t remember	28 (4.9)	11 (4.1)	17 (5.6)	
Reason for taking the last course of antibiotics	Pneumonia	36 (6.3)	15 (5.5)	21 (6.9)	0.001
	Bronchitis	65 (11.3)	35 (12.9)	30 (9.9)	
	Rhinopharyngitis	61 (10.6)	22 (8.1)	39 (12.9)	
	Flu	126 (22.0)	71 (26.2)	55 (18.2)	
	Cold	199 (34.7)	92 (33.9)	107 (35.3)	
	Sore throat	147 (25.6)	68 (25.1)	79 (26.1)	
	Cough	144 (25.1)	57 (21.0)	87 (28.7)	
	Fever	151 (26.3)	61 (22.5)	90 (29.7)	
	Headache	107 (18.6)	38 (14.0)	69 (22.8)	
	Diarrhea	13 (2.3)	6 (2.2)	7 (2.3)	
	Urinary tract infection	38 (6.6)	28 (10.3)	10 (3.3)	
	Skin or wound infection	25 (4.4)	7 (2.6)	18 (5.9)	
	Other	90 (15.7)	44 (16.2)	46 (15.2)	
	I do not wish to answer	11 (1.9)	3 (1.1)	8 (2.6)	
	I do not know	1 (0.2)	0 (0.0)	1 (0.3)	
	COVID-19	15 (2.6)	8 (3.0)	7 (2.3)	
Consumption of antibiotics in case of COVID-19 infection	I did not get COVID-19	453 (41.1)	249 (45.0)	204 (37.1)	0.001
	I did not take antibiotics	235 (21.3)	110 (19.9)	125 (22.7)	
	From a medical prescription	168 (15.2)	76 (13.7)	92 (16.7)	
	Administered by a medical practitioner	71 (6.4)	41 (7.4)	30 (5.5)	
	Use of leftover antibiotics	49 (4.4)	29 (5.2)	20 (3.6)	
	Without prescription from a pharmacy	82 (7.4)	40 (7.2)	42 (7.7)	
	Without prescription from elsewhere	44 (4.0)	16 (2.9)	28 (5.1)	
	I don’t remember	87 (7.9)	30 (5.4)	57 (10.4)	
	I do not wish to answer	18 (1.6)	5 (0.9)	13 (2.4)	
	I don’t know	30 (2.7)	20 (3.6)	10 (1.8)	
Impact of the COVID-19 pandemic on the need for antibiotics (multiple answers were permitted)	The need to take antibiotics decreased due to strengthened personal protective measures	351 (31.8)	190 (34.4)	161 (29.3)	0.027
	The need to take antibiotics decreased due to falling ill less often during the containment period	234 (21.2)	120 (21.7)	114 (20.7)	
	Access to antibiotics was restricted due to the inability to visit a doctor for a prescription	57 (5.2)	28 (5.1)	29 (5.3)	
	Access to antibiotics was restricted due to the inability to visit a pharmacy	41 (3.7)	20 (3.6)	21 (3.8)	
	The need to take antibiotics increased	116 (10.5)	53 (9.6)	63 (11.5)	
	Access to antibiotics remained the same	324 (29.4)	165 (29.8)	159 (28.9)	
	I don’t remember	118 (10.7)	43 (7.8)	75 (13.6)	
	I do not wish to answer	32 (2.9)	11 (2.0)	21 (3.8)	
	I don’t know	137 (12.4)	59 (10.7)	78 (14.2)	

* Pearson’s chi-squared test was used to compare the differences between 2022 and 2024.

**Table 5:** Antibiotic consumption in defined daily doses (DDDs) per 1000 inhabitants per day and packages per inhabitant per year.

Antibiotics			Defined Daily Doses per 1000 Inhabitants		Packages per Inhabitant per Year	
ATC5 Code	Substance	Pharmacological Group	2021	2023	2021	2023
J01AA02	Doxycycline	Tetracyclines	1.132178	1.52923	0.041325	0.055304
J01AA07	Tetracycline		0.191866	0.193768	0.035015	0.035363
J01BA01	Chloramphenicol	Amphenicols	0.750606	0.733785	0.164697	0.161012
J01BA02	Thiamphenicol		0.021473	0.037614	0.007838	0.013729
J01CA01	Ampicillin	Penicillins	0.661774	0.593473	0.263383	0.211687
J01CA04	Amoxicillin		1.506687	1.443317	0.16913	0.13585
J01CE01	Benzylpenicillin		0.052307	0.045701	0.06461	0.054724
J01CE08	Benzathine benzylpenicillin		0.020275	0.018901	0.033271	0.026842
J01CR01	Ampicillin and beta-lactamase inhibitor	Beta-lactam	0.000114	5.63756 × 10^−5^	0.000501	0.000247
J01CR02	Amoxicillin and beta-lactamase inhibitor		1.553602	2.236595	0.121446	0.165961
J01CR04	Sultamicillin		0.0	0.000178	0.0	2.170769 × 10^−5^
J01CR05	Piperacillin and beta-lactamase inhibitor		0.001085	0.000605	0.001246	0.000773
J01DB01	Cefalexin	First-generation cephalosporins	0.002023	0.0	0.000591	0.0
J01DB04	Cefazolin		0.718678	0.595132	0.789158	0.616947
J01DC02	Cefuroxime	Second-generation cephalosporins	0.986687	1.045222	0.141141	0.130789
J01DC10	Cefprozil		0.028921	0.106499	0.002035	0.007352
J01DD01	Cefotaxime	Third-generation cephalosporins	1.732240 × 10^−5^	0.0	2.529070 × 10^−5^	0.0
J01DD02	Ceftazidime		0.045161	0.023554	0.057793	0.027337
J01DD04	Ceftriaxone		2.571002	2.037079	1.873313	1.45889
J01DD08	Cefixime		0.237291	0.342224	0.024092	0.036496
J01DD12	Cefoperazone		0.00556	4.181704 × 10^−5^	0.002624	4.477211 × 10^−5^
J01DD13	Cefpodoxime		0.096825	0.107968	0.00504	0.004704
J01DD15	Cefdinir		0.013421	0.02393	0.00098	0.002869
J01DD52	Ceftazidime and beta-lactamase inhibitor		0.000221	0.0	0.000242	0.0
J01DD62	Cefoperazone and beta-lactamase inhibitor	Beta-lactam	3.464480 × 10^−7^	0.001773	5.058141 × 10^−7^	0.002588
J01DE01	Cefepime	Fourth-generation cephalosporins	0.026631	0.02017	0.038203	0.02854
J01DH02	Meropenem	Carbapenems	0.016121	0.015709	0.012668	0.006991
J01DH03	Ertapenem		0.000162	0.0	5.918025 × 10^−5^	0.0
J01DH04	Doripenem		3.787831 × 10^−5^	2.478047 × 10^−6^	4.147676 × 10^−5^	2.713461 × 10^−6^
J01DH51	Imipenem and cilastatin		0.000171	0	0.000329	0
J01DI02	Ceftaroline fosamil	Fifth-generation cephalosporins	0.001158	4.130078 × 10^−5^	0.000507	1.808974 × 10^−5^
J01EB01	Sulfaisodimidine	Sulfonamides	0.449555	0.312369	0.218783	0.152019
J01EE01	Sulfamethoxazole and trimethoprim	Sulfonamide–trimethoprim combinations	0.647122	0.64411	0.060283	0.055384
J01FA01	Erythromycin	Macrolides	0.1102	0.083102	0.022728	0.012131
J01FA02	Spiramycin		0.155473	0.13279	0.024795	0.020757
J01FA03	Midecamycin		0.051662	0.022773	0.004215	0.001584
J01FA06	Roxithromycin		0.041532	0.03784	0.003032	0.002762
J01FA07	Josamycin		0.013013	4.336582 × 10^−5^	0.001085	3.617948 × 10^−6^
J01FA09	Clarithromycin		0.717991	0.849987	0.025572	0.030169
J01FA10	Azithromycin		3.455283	3.336897	0.306333	0.287165
J01FF01	Clindamycin	Lincosamides	0.014443	0.011151	0.001548	0.001165
J01FF02	Lincomycin		0.059723	0.046895	0.016596	0.018239
J01GA01	Streptomycin	Aminoglycosides	0.005352	0.010773	0.001136	0.002182
J01GB03	Gentamicin		0.064832	0.177614	0.00142	0.00389
J01GB06	Amikacin		0.042727	0.034813	0.033105	0.025107
J01MA01	Ofloxacin	Fluoroquinolons	0.158856	0.157443	0.035704	0.047447
J01MA02	Ciprofloxacin		2.295927	2.348062	0.214517	0.219895
J01MA06	Norfloxacin		0.117127	0.060594	0.006636	0.003272
J01MA07	Lomefloxacin		0.00327	0.002013	0.000239	0.000147
J01MA12	Levofloxacin		0.974015	0.830394	0.120358	0.082017
J01MA14	Moxifloxacin		0.039458	0.044832	0.003283	0.002598
J01MB04	Pipemidic acid	Quinolones	6.92896 × 10^−5^	0.0	5.058141 × 10^−6^	0.0
J01RA09	Ofloxacin and ornidazole	Combined medications	0.0	0.0	0.000246	0.0
J01RA12	Ciprofloxacin and ornidazole		0.0	0.0	7.587212 × 10^−6^	0.0
J01XA01	Vancomycin	Glycopeptides	0.000204	3.840972 × 10^−5^	0.000149	2.803910 × 10^−5^
J01XB01	Colistin	Polymyxins	0.0	8.397825 × 10^−5^	0.0	2.713461 × 10^−5^
J01XD01	Metronidazole	Imidazoles	0.55975	0.688165	0.614425	0.716337
J01XD02	Tinidazole		0.0	0.0	1.972675 × 10^−5^	0.0
J01XD03	Ornidazole		1.040037 × 10^−5^	1.396999 × 10^−5^	0.000759	0.00102
J01XE01	Nitrofurantoin	Nitrofuran derivates	0.516408	0.534605	0.075396	0.077706
J01XE03	Furazidin		0.665076	0.669819	0.04995	0.048658
J01XX01	Fosfomycin	Phosphonics	0.098063	0.147554	0.030828	0.045439
J01XX04	Spectinomycin	Imidazoles	0.000129	0.0	7.081397 × 10^−5^	0.0
J01XX07	Nitroxoline	Nitroquinolines	0.3185	0.211758	0.046501	0.030917
J01XX08	linezolid	Oxazolidinones	2.249602 × 10^−6^	1.009804 × 10^−6^	0.000493	0.000221
Total			22.21783	22.5491	5.771523	5.073366

## Data Availability

The data presented in this study are available on request from the corresponding author. Due to the personal nature of the data, they are not publicly accessible.
